# Poly (ADP-ribose) polymerase 1 (PARP1) inhibition promotes pulmonary metastasis of osteosarcoma by boosting ezrin phosphorylation

**DOI:** 10.7150/ijbs.58784

**Published:** 2022-01-09

**Authors:** Fangfei Li, Xiaoqiu Wu, Xuekun Fu, Jin Liu, Wangze Song, Gary Guishan Xiao, Aiping Lu, Ge Zhang

**Affiliations:** 1 Shum Yiu Foon Sum Bik Chuen Memorial Centre for Cancer and Inflammation Research (CCIR), School of Chinese Medicine, Hong Kong Baptist University, 999077, Hong Kong SAR, China.; 2 Law Sau Fai Institute for Advancing Translational Medicine in Bone & Joint Diseases, Hong Kong Baptist University, 999077, Hong Kong SAR, China.; 3 Guangdong-Hong Kong-Macau Joint Lab on Chinese Medicine and Immune Disease Research, 999077, Hong Kong SAR, China.; 4 Department of Biology, Southern University of Science and Technology, Shenzhen 518055, China.; 5 Department of Pharmacology, School of Chemical Engineering, Dalian University of Technology, 116024, Dalian, Liaoning, China.; 6 Institute of Basic Research in Clinical Medicine, China Academy of Chinese Medical Sciences, 100700, Beijing, China.; 7 Institute of Arthritis Research, Shanghai Academy of Chinese Medical Sciences, 200032, Shanghai, China.

**Keywords:** Osteosarcoma, metastasis, PARP1, ezrin, combination therapy.

## Abstract

Due to the large proportion of BRCA deficiency and chromosomal instability in OS patients, poly (ADP-ribose) polymerase inhibitors (PARPi) could be an effective strategy for anti-OS therapy. In two orthotopic OS mouse models, we discovered that although PARPi had inhibitory effect on the growth of the orthotopic OS tumors regardless of BRCA deficiency, the treatment of PARPi essentially aggravated the pulmonary metastasis of OS in both models. A protein playing a crucial role in OS metastasis, ezrin, was identified as an interactive protein for PARP1. The phosphorylation of ezrin was significantly promoted during PARP inhibition. Besides the traditional function of phosphorylated ezrin at plasma membrane, we newly identified its nuclear speckle localization and its function with mRNA export. Ezrin knockdown or phosphorylation inhibition could partially rescue PARPi induced metastasis. Collectively, we unveiled a new mechanism for PARP-involved OS metastasis, which proposed a novel combinational therapy strategy using PARP and ezrin inhibitors for future OS treatment.

## Introduction

Osteosarcoma (OS) is a primary bone sarcoma mostly occurring in children and adolescence 1, 2. The frequent complications of OS are rapid progression and pulmonary metastases 3, 4. Effective treatment options are still lacking for OS patients with pulmonary metastases and recurrent disease 5, 6. Genome instability and a large proportion of BRCA1/2 mutations have been found occurring in OS oncogenesis 7, 8. Generally, in patients carrying germline or somatic defects in homologous-repair (HR) genes like BRCA1/2, DNA repair is heavily dependent on other DNA repair related genes, such as Poly (ADP-ribose) polymerase 1 (PARP1) 7, 9, 10. Therefore, the inhibition of PARP1 could be a promising strategy for OS treatment.

Poly (ADP-ribose) polymerases (PARPs) are a family of enzymes regulating DNA repair and genome transcription 11-13. They post-translationally modify proteins by attaching ADP-ribose polymers (or poly ADP-ribose, PAR) on certain domains using NAD as a substrate, which process is termed as PARylation. PARylation had been found occurring in a wide range of intracellular proteins and played crucial roles in diverse biological functions. Besides the mostly focused area in chromatin regulation, transcription, and DNA repair, PARPs are also found linked to stress responses, metabolism, and cancer 12. PARP inhibitors bind to the NAD binding pocket in the catalytic domain of PARPs and hinder the synthesis of PAR 11-13. PARP1 contributes to over 90% of PAR synthesis and is considered as the major target for PARP inhibitors 14. The PARP inhibitors have been approved for the clinical treatment of metastatic and recurrent ovarian and breast cancer patients with BRCA1/2 mutations by FDA, and there are a number of on-going clinical trials for treating other types of cancers with PARPi 15, 16.

Epithelial-mesenchymal transition (EMT) is a molecular program closely associated with cancer metastasis. During EMT, cancer cells lose their cell-cell adhesion properties and acquire invasive and migratory properties, which is presented by decreased expression of epithelial markers (e.g. E-cadherin) and increased expression of mesenchymal markers (e.g. vimentin) 17. Though EMT mostly refers to cancers of epithelial origin, tumors of mesenchymal origin such as osteosarcoma are found also expressing epithelial markers and could undergo EMT process upon stimulations 18, 19.

Ezrin is a protein closely associated with metastasis in OS 20-22. Ezrin belongs to the ezrin-radixin-moesin (ERM) family and mainly found localizes on plasma membrane 20, 21. It has been established that the phosphorylation of ezrin on its C-ERMAD (C-terminal ERM-association) domain promotes cell mobility and metastasis by crosslinking actin cytoskeleton and plasma membrane 20, 21. Ezrin was reported highly expressed in OS metastatic tissues and there were compelling evidences for a metastasis-promoting function of ezrin in osteosarcoma and other sarcoma subtypes 20, 21.

In this work, using orthotopic osteosarcoma mouse models and osteosarcoma cell lines, we unexpected discovered that although PARPi could efficiently reduce tumor growth, the inhibition of PARP essentially promoted the metastasis of osteosarcoma. We discovered that PARP1 interacted with a protein playing a crucial role in osteosarcoma metastasis - ezrin and regulated its phosphorylation. Beyond the traditional findings - the phosphorylated ezrin localizes at cell membrane and promotes cell motion by interacting with F-actin, we newly discovered that a portion of p-Ezrin located in the nuclear speckles and participated in RNA export which affected EMT. The combination of the PARP inhibitor olaparib with an ezrin inhibitor dramatically inhibited OS growth with reduced PARPi induced OS metastasis.

## Materials and Methods

### Cell lines

K7M2 (ATCC® CRL-2836™) and 143B (ATCC® CRL-8303™), U2OS (ATCC® HTB-96™) and MG-63 (ATCC® CRL-1427™) were osteosarcoma cell lines. K7M2 was maintained in ATCC-formulated Dulbecco's Modified Eagle's Medium (DMEM, Catalog No. 30-2002) supplemented with 10% fetal bovine serum (FBS, Thermo Fisher Scientific) and 100 U/mL Penicillin-Streptomycin (PS). U2OS and MG63 were cultured in DMEM supplemented with 10% FBS and 100 units/mL PS. 143B cells were cultured in minimum essential medium (Eagle) in Earle's BSS (EMEM) supplemented with 10% FBS, 0.015 mg/ mL 5-bromo-2'-deoxyuridine and 100 U/mL PS. All cells were cultured under normal culture conditions with 95% humidity and 5% CO2 at 37 °C. All the cells used for experiments were tested negative for bacteria, yeast, fungi and mycoplasma.

### Reagents and antibodies

Olaparib, Talazoparib (BMN 673) and NMS-P118 were obtained from Selleck Chemicals (Houston, TX, USA) and dissolved in dimethyl sulfoxide (DMSO). NSC668394 was obtained from MERK (Merck Millipore, Molsheim, France) and dissolved in DMSO. His-tagged HSA protein and his-tagged PARP1 protein were purchased from Sino Biological Inc. Cell Navigator™ Live Cell RNA Imaging Kit *Green Fluorescence* was obtained from AAT Bioquest.

The flowing primary antibodies were used in this work: Anti-Ezrin (phospho T567) polyclonal antibody (Abcam, ab47293), anti-ezrin monoclonal antibody (Abcam, ab40839), anti-PARP1 monoclonal antibody (Abcam, ab32138), anti-β-actin monoclonal antibody (Abcam, Abcam 8226), anti-Poly (ADP-Ribose) polymer polyclonal antibody (Abcam, ab14460), Anti-Cortactin antibody [4F11] (Abcam, ab33333), E-cadherin polyclonal antibody (Proteintech, 20874-1-AP), fibronectin polyclonal antibody (Proteintech, 15613-1-AP), vimentin polyclonal antibody (Proteintech, 10366-1-AP), and anti-MMP2 antibody (Abcam, ab97779), anti-FLAG polyclonal antibody (Abcam, ab1162), anti-Lamin B1 antibody (Abcam, ab16048), PKC Iota polyclonal antibody (Proteintech, 13883-1-AP), ROCK1 polyclonal antibody (Proteintech, 21850-1-AP), ROCK2 monoclonal antibody (Proteintech, 66633-1-Ig), Anti-PI3 Kinase p85 alpha (phospho Y607) antibody (Abcam, ab182651).

### Western blotting

Whole proteins or proteins in different components from treated OS cells were extracted using ice-cold RIPA lysis buffer supplemented with 1X Protease and Phosphatase Inhibitor Cocktail. The protein concentrations were determined by Pierce™ BCA Protein Assay Kit, referred to the manufacture's guidelines. Then 30 μg protein in each group were prepared using 5X SDS PAGE protein loading buffer and denatured at 100 °C for 10 min. After centrifugation, loaded equal amounts of protein into the lanes of the 10% SDS-PAGE gel, respectively. In order to show the protein band, 10 μL of molecular weight marker was also loaded into an empty lane. After running for 1.5 h at 110 V in running buffer, the polyvinylidene difluoride (PVDF) membranes (Millipore) was activated by incubating with methanol for 1 min before using. Then the proteins in the SDS-PAGE gel were transferred to the activated PVDF membrane through running in the transferring buffer for another 1.5 h at 300 mA in an ice box. After transferring, based on the protein marker the protein bands were cut flowed by washing with TBS-T (20 mM Tris-HCl, pH 7.5, 150 mM NaCl, and 0.5% Tween 20). Then the protein bands were blocked using 5% milk or 5% BSA for 1h at room temperature. Then incubated the membranes with suitable dilutions of primary antibodies in blocking buffer for overnight. After washing with TBS-T for three times, 15 min for each time, the protein bands were incubated with the recommended dilution of horseradish peroxidase (HRP)-labeled secondary antibodies based on the host species of primary antibodies at room temperature for 1.5 h. At last, enhanced chemiluminescence (ECL) Western Blotting Substrate was used for visualizing the expression of the target proteins. The expression level of the target proteins was quantified and normalized to corresponding loading control protein 23.

### Real-time quantitative PCR analysis (RT-qPCR)

To evaluate whether PARP inhibitor induced metastasis was related to EMT, 143B cells and K7M2 cells were treated with olaparib or vehicle for 48 h, respectively. And the mRNA level of EMT markers and transcriptional factors were measured through RT-qPCR. RT-qPCR was carried out as previously described 24. In brief, OS cells treated with or without olaparib were washed with ice-cold PBS and added 1 mL TRIzol reagent to extract total RNA. RNA in cytosol or nucleus was isolated using Cytoplasmic and Nuclear RNA Purification Kit. The concentration of isolated RNA was determined spectrophotometrically and finally adjusted to 1 μg for the reverse transcription (RT) step by using the High-Capacity RNA-to-cDNA™ Kit. SYBR® Select Master Mix was used to prepare the PCR reaction mix, according to the user guide. Four replicates of each reaction were performed to get the best accurate result. Briefly, prepared the appropriate number of reactions firstly. Then aliquoted 20 μL of the PCR mix to each well of the RT-qPCR plates. After sealing the plates, centrifuged at 1,000 rpm to spin down the sample to the bottom of the tubes and eliminate any air bubbles. ABI 7500 thermal cycler (Applied Biosystems) was used and the application process sets as flows: 1 min at 94 °C for one cycle, followed by 42 cycles for 30 sec for 94 °C, 30 sec for 60 °C and 1 min for 72 °C. All employed primer sequences in this work were summarized in **Supplementary Table 1**. The fluorescence signal of SYBR was converted into numerical values by SDS 2.1 software. And the relative expression levels of mRNA of the target genes were normalized to the internal control 25.

### Colony formation assay

In order to evaluate the effect of olaparib on the proliferation ability in OS cells, we conducted colony formation assay according to a previously described method 26. Briefly, single cell suspensions of 143B or K7M2 cells were seeded and maintained in 6-well plates (1,000 cells each well) in according complete medium for overnight. And then changed the medium with conditioned medium supplemented with DMSO, or olaparib, the conditioned medium was changed every two days. After treatment for 10 days at cell incubator, washed the cells with cold PBS for three times and fixed the surviving proliferating clones with 4% paraformaldehyde for 15 min in dark flowed by staining with 0.5% crystal violet staining solution (Beyotime Biotechnology) for 0.5 h. Then removed the crystal violet carefully and rinsed the cells with tap water. The numbers of clones were photographed.

### siRNA interference

siRNA sequences that targeted the human PARP1, PARP2, ezrin were purchased from Guangzhou RiboBio Co., LTD. We prepared siRNA sequences according to the user guide. A non-specific sequence with no homology with the human genome was used as a control sequence. The human osteosarcoma 143B cells had been seeded in a 6-well plate with complete medium and incubated 24 h. On the next day, when growing to 80% confluence, the culture medium of the 143B cells at logarithmic growth phase were removed and replenished with 1.7 mL fresh complete medium. 25 pmol PARP1, PARP2 siRNA or empty vector with 7.5 μL lipofectamine™ RNAiMAX transfection reagent per well was transfected for 48 h in 143B cells. Then the cells were harvested, some cells were used for wound healing assay, transwell assay and others used to extract protein for western blot analysis. The sequences of siRNAs used in this study are shown in **Supplementary Table 2**.

### Invasion assay

To investigate the effect of PARP inhibiters, EZRi and PARP1 on the invasion ability of OS cells* in vitro*, 143B cells and K7M2 cells were seeded 24 h in advance flowed by treating with DMSO, PARP inhibitors, EZRi, EZRi combined with olaparib, or transfected with siRNA. After treatment, the cells were detached with Accutase to prepare single cell suspensions. After washing two times with serum free medium, counted the cells and prepared single cell suspensions containing 5.0 × 10^5^ cells/mL in medium without serum but supplemented with DMSO or PARP inhibitors. The invasion ability of treated cells was performed by 24-Multiwell Insert System (8.0 μm PET membrane, Corning). 800 µL full medium was firstly added into the wells and then put in the inserts. 200 µL of cell suspensions were transferred into the upper chambers and incubated for 10 h at cell incubator. Ten hours later, removed the inserts into new 24-well plates and washed the cells with PBS for three times and then fixed the cells. After three washes with ddH2O, fixed the cells with 4% paraformaldehyde for 15 min in dark flowed by staining using 0.5% crystal violet staining solution for 0.5 h. After three washes with ddH2O, the uninvaded cells were gently scraped with cotton swabs. The invaded cells on the lower side of the filter membrane were observed at approximately 40X and imaged using a brightfield microscopy. The number of invaded cells in 15 randomly fields were counted for statistics.

### Wound healing assay

To investigate the effect of PARP inhibiters, EZRi and PARP1 on the invasion ability of OS cells *in vitro*, 143B cells and K7M2 cells were seeded 24 h in advance flowed by treating with DMSO, PARP inhibitors, EZRi, EZRi combined with olaparib, or transfected with siRNA. Then the cells were detached with Accutase to prepare single cell suspensions. Then transferred the treated 143B cells or K7M2 cells into 6-well plates for overnight until a confluent monolayer of cells formed. Uniform wounds were carefully made by scraping the cell plates in a "#" glyph using a 10 μL pipette tip. PBS was used to wash off the floating cells, complete culture medium supplemented with DMSO or PARP inhibitors were added to the plates. The images captured at 10X magnification by an inverted microscope at this time was defined 0 h as the baseline. Then incubated the cells at incubator for another 10 h and flowed by photograph. The scratch area at 0 h was set to 100%. The migration rate was analyzed using ImageJ software 27.

### Adhesion assay

To investigate whether olaparib exhibits an effect on the adhesion ability of OS cells, 143B cells and K7M2 cells were seeded 24 h in advance flowed by treating with DMSO or olaparib, respectively. The adhesion ability was assessed using the CytoSelect™ Cell Adhesion Assay Kit according to the user manual 28. In brief, 3.0× 105 143B or K7M2 cells treated by PAPR inhibitors were seeded in the well with 150 μL serum free medium and incubated for 1 h at cell incubator. Then gently discarded the medium and washed PBS for 5 times with 250 µL for each time. Added 200 μL of Cell Stain Solution supplied by the kit to each well and incubated for 0.5 h. Then gently discarded the medium and washed 5 times with 250 µL ddH2O for each time. When the wells were dry, the images could be captured. Then added 200 μL of Extraction Solution to each well to incubate for 0.5 h on a rocking shaker. Aspirated 150 µL of the mixture solution to a new 96-well plate. The absorbance at 560 nm was measured by an enzyme immunoassay analyzer. The adhesion ability was quantified by comparison with the control BSA group.

### X-Ray analysis

In order to determine the tumor size, the mice were anesthetized and digital radiography of the tumor bearing mice were made using a Faxitron MX-20 X-Ray equipment each week. The width and length were measured. The tumor size was calculated according to the formula: volume = (width)^2^ x length/2 29, 30.

### Biophotonic imaging analysis

After corresponding treatment, the mice were sacrificed, and tumor tissues and lung tissues were isolated. Due to 143B cells was modified with mCherry modification, therefore, the tumor tissues and lung tissues could be visualized through detecting the signal of mCherry using an IVIS® Lumina XR imaging system (Xenogen Imaging Technologies). The detailed parameters were referred to our previous published work 30.

### Co-immunoprecipitation (co-IP)

Co-IP assay was conducted with Pierce™ Classic Magnetic IP/co-IP Kit referring to the protocol for user 31, 32. Briefly, 143B cells were cultivated in 10 cm dishes 24 h in advance. Then detached the cells by incubating with Accutase for 3 min at cell incubator. After centrifugation and three washes using PBS, 1 mL of IP Lysis/Wash Buffer provided by the kit was added to the pellet for incubating for 5 min on ice with periodic mixing. After centrifugation, transferred equal volume of the supernatant into two fresh microcentrifugation tubes. 5 μg IP antibodies for PARP1 or IgG were added and incubated overnight on a spinning wheel at 4 ºC to sufficiently form the immune complex. After incubation, the immune complex was incubated with magnetic protein A/G beads for 1 h. After three washes with lysis buffer, 2X SDS PAGE protein loading buffer was added to the proteins-beads complex to denature at 100 °C for 10 min on a heating block. Then, immunoprecipitants were analyzed using SDS-PAGE gel flowed by staining with Pierce™ Silver Stain.

### Pull-down assay

To identify the target substrate protein of PARP1, a pull-down assay was performed as indicated: briefly, PARP1 protein with his-tag, or human serum albumin (HSA) protein with his-tag were incubated with Ni-NTA Magnetic Agarose Beads (Qiagen) on a spinning wheel at cold room for 2 h to mobilize the proteins on beads. After mobilization, washed the proteins-beads complex three times with PBS buffer containing protease inhibitors. Then, the protein-beads complex was incubated with the whole proteins of 143B prepared as mentioned above for overnight at cold room. After washing, the pull-down complexes were denatured at 100 °C on a heating block for 10 min to isolate proteins from the beads. The supernatant contains the proteins was collected and separated using SDS-PAGE gel and visualized through silver staining 32.

### Immunofluorescence staining of cultured cells

To show the subcellular distribution of p-Ezrin, PARP1 in 143B cells, 1.5 x 10^5^ 143B cells were seeded in Nunc glass bottom dishes 24 h in advance. On day 2, fixed the cells using 4% PFA in dark for 15 min. After washing four times with PBS, permeabilized the cells with 0.5% Triton X-100 for 15 min. Then, blocking buffer which contains 1% BSA and 2% goat serum was added to each well and incubated for 1 h. After blocking, incubated primary antibodies for ezrin, p-Ezrin, SC-35, PARP1 at appropriate dilution concentration with the cells for 1 h, respectively. After four washes with PBS, fluorescent conjugated secondary antibodies were added and incubated for 1 h. The nuclei were counterstained using 1mg/mL Hoechst 33342 (Beyotime, Catalog number: C1027) and visualized using a Leica confocal microscope 33, 34.

### RNA imaging

To demonstrate olaparib induced RNA export was dependent on p-Ezrin, 1.5 x 105 143B cells were seeded in Nunc glass bottom dishes 24 h in advance. Then the log-phase cells were treated with DMSO, olaparib, ezrin inhibitor, or the combination of olaparib and ezrin inhibitor, respectively. After fixation and permeabilization, the subcellular localization of RNA was shown by staining the cells with 1X Cell Navigator™ Live Cell RNA Imaging Kit *Green Fluorescence* (AAT Bioquest) solution diluted with PBS for 0.5 h at room temperature. The fluorescence intensity of RNA-488 was monitored by Leica confocal microscope.

### Stability of mRNA

To understand the effect of olaparib on mRNA stability, 143B cells were seeded 24 h in advance flowed by treating with DMSO or olaparib, respectively. At the end of treatment, transcription inhibitor Act D was added to the medium and incubated for 0, 1, 2, 3 and 4 h, respectively. The remaining RNA of E-cadherin, vimentin, fibronectin and MMP2 was measured using RT-qPCR as described above.

### Immunofluorescence staining of lung sections

In order to demonstrate the pro-metastasis function of olaparib in NOD/SCID gamma mice, and EZRi could rescue olaparib induced metastasis, the lung tissues from treated mice were isolated for preparing cryo-sections. In brief, lung tissues were fixed using 4% paraformaldehyde at least for 48 h on a rocking shaker, followed by equilibration in gradient concentrations of sucrose solution (15%, 20%, 30%) for 24 h, respectively. After equilibration, the tissues were embedded using optimal cutting temperature (O.C.T) compound (Sakura Finetek USA, Catalog Number: 4583) and liquid nitrogen. These tissue blocks could be stored at -80 °C. Then 5 μm sections were prepared and the nuclei were counterstained using 1mg/mL Hoechst 33342. The fluorescence intensity of mCherry was examined under a Leica confocal microscope.

### Quantification of circulating cancer cells in blood via flow cytometry sorting

In order to quantify the percentage of the circulating cancer cells in the blood obtained from NOD/SCID mice treated with vehicle, olaparib, EZRi, olaparib combined with EZRi, fresh whole blood was taken from hearts of deeply anaesthetized mice by cardiac puncture. In brief, to minimize hair flying around, 70% ethanol solution was used to wet down and disinfect the fur. 21‐gauge needles, 1 mL syringes and blood collection microtubes were recoated with fresh 0.5 % w/v heparin for anticoagulation. 250 μL whole blood sample in each group was used and red blood cells were lysed by RBC Lysis Buffer (Biolegend) according to the user manual. mCherry positive cancer cells in each group were sorted via a FACS Aria III apparatus (BD Biosciences) 35, 36.

### Homology modeling of ezrin and molecular docking with PARP1

Homology modeling of EZRIN was based on X-ray crystal structure of moesin (PDB ID: 2i1j) and EZRIN FERM and C-terminal domain. 3D structure of EZRIN is available in PDB database, however only FERM and C-terminal domain are resolved (Structural characterization suggests models for monomeric and dimeric forms of full-length ezrin). Blast (http://blast.ncbi.nlm.nih.gov/Blast.cgi) against PDB database was performed to identify homology templates, moesin from spodoptera frugiperda is identified as the only available structure that has full length alignment except EZRIN itself. Full length structure prediction was based on the available partial structure of EZRIN itself (PDB ID: 4RM8) and moesin (PDB ID: 2i1j). Homology modeling software MODELER was used to build 3D structure of EZRIN. Structure with best DOPE score (Discrete Optimized Protein Energy) was chosen as the starting point for further structure refinements. In order to find optimized conformations, loop refinement was applied to the loop region residues of the predicted structure of model protein. After loop refinement, energy minimization was applied to the model structure to remove obvious clashes.

Two steps were used to get final possible docking structures of PARP1 and EZRIN. ZDOCK: a fast, rigid-body protein-protein docking algorithm that applies ligand rotation and a pair-wise shape complementarily. The X-Ray structure of PARP1 is used as receptor and predicted structure of EZRIN is used as ligand in the ZDOCK, angle sampling size of 15 degrees were used to search the docking poses. RMSD cutoff 10.0Å were used to cluster poses, and the maximum number of 100 clusters would be generated. RDOCK: based on the CHARMm simulations program and used for further refinement of complexes generated by ZDOCK, it ranks the docked structures based on CHARMm electrostatic interaction energy and ACE desolvation energy. Two poses that have either have good ZDock score or cluster density from top 6 clusters were used here to further refine of the docking structure using Rdock. The optimal model was finally selected.

### Treatment of mice

To evaluate the anti-tumor and anti-metastatic effects of the PARP inhibitor olaparib, two orthotopic OS mouse models were established according to previous reports 30. Briefly, NOD/SCID gamma mice (or BALB/c mice) were anesthetized. The knee of the right hind limb was flexed beyond 90° and a 25-gauge needle was used to rotate and penetrate the proximal tibial crest cortex. 20 μL of 143B-mCherry cells, ezrin^KD^, ezrin^KD+WT^, ezrin^KD+Mut^ (or K7M2 cells) suspension with around 1.0 x10^6^ cells were injected into each mouse. The mice were monitored for the appearance of tumor growth. Three weeks after inoculation, the mice with observed tumor on the right hind limb were selected for PARP inhibitor evaluation studies.

According to the previously reported studies and our *in vitro* results on olaparib, the dose of olaparib was set as 100 mg/kg intraperitoneally once daily for five weeks in both mouse models. After five weeks dosing, 15 mice of each group were deeply anaesthetized and the orthotopic tumor were imaged by X-Ray. Blood was collected by cardiac puncture and stored in EDTA-containing tubes. The visible metastatic foci in lung tissue was counted and the lung tissue was divided into two portions: one portion was digested into single cell suspension for the analysis of the micro-metastasis in lung by calculating the percentage of mCh^+^ in lung by flow cytometry; another portion was fixed by 4% paraformaldehyde and sectioned for imaging by confocal microscopy.

To evaluate whether EZRi could rescue olaparib induced lung metastasis *in vivo*, the same two mouse models were used. After inoculation for three weeks, equal number of mice were treated with vehicle, olaparib, EZRi, olaparib combined with EZRi, respectively. Five weeks later, 15 mice of each group were deeply anaesthetized. Then the tumor volume, tumor weight, the percentage of CTCs in the circulating system, the visible lung metastatic nodules counts, and the percentage of mCh^+^ cells in lung tissue were evaluated using the method as described above.

### Pulmonary metastasis analysis

In order to evaluate the lung metastasis in BALB/c mice after olaparib treatment, the lung tissues were used for preparing 5-μm-thick sections. These tissues were embedded with paraffin for further H&E staining. The visible nodules on lung tissues were counted. To analyze the lung metastasis in NOD/SCID gamma mice after treatment, the lung tissues were used for preparing 5-μm-thick sections and counterstained with DAPI. The mCherry and DAPI fluorescence were measured by confocal imaging. The visible nodules on lung tissues were also counted.

### Hematoxylin/eosin (H&E) staining

The H&E experiments were conducted according to previous published work 37. The procedures contained (1) remove the wax; (2) hydrate the section; (3) nuclear staining and blueing; (4) differentiation; (5) eosin counterstain; (6) dehydrate; and (7) mount. To remove wax, paraffin-embedded lung tissues isolated from BALB/c mice treated with vehicle, EZRi, olaparib, EZRi and olaparib were incubated with xylene for 15 min each for two times. Process for hydration included 100% ethanol for 2 min, twice; 95% ethanol for 3 min, twice; 80% ethanol for 3 min; 70% ethanol for 3 min. Then rinsed sections in small flow water for 5 min. After washing, stained the tissue sections with hematoxylin staining for 10 min to stain the nucleus. After washing with tap water, transferred the paraffin slices into 1% hydrochloric acid ethanol differentiation liquid solution in 5~30 seconds until the slice get red, then rinse water for about 10 min to the section of the eye can be seen blue. Incubated 1% eosin solution with the slides for 12 min, flowed by washing for 1 min with water. Then paraffin slices were put into 70% ethanol for 2 min; 80% ethanol for 2 min; 95% ethanol for 2 min, twice; 100% ethanol for 2 min for twice to dehydrate. Then paraffin slices were put into xylene I solution and xylene II solution each for 10 min to clear the tissue slides and make it completely transparent; After air dried the slices and sealed the slices with neutral Canada gum and covered with a glass cover slip.

### Statistical analysis and reproducibility

For most *in vitro* studies, the statistical analysis of results was presented based on mean ± standard deviation unless otherwise noted. In order to ensure the reliability of the data, the *in vitro* experiments were performed at least three times independently. For *in vivo* experiments, the results were analyzed using Prism - GraphPad and each dot in the graph represents one mouse. The representative images we chose and showed in this research work was dependent on the average/median level of the data for each group. Generally, we used one-way analysis of variance (ANOVA) with a Tukey's multiple comparisons test to define the differences in treated groups. P value lower than 0.05 was considered statistically significant.

## Results

### Olaparib promoted pulmonary metastasis of osteosarcoma in tumor bearing mouse models

**Figure 1:**
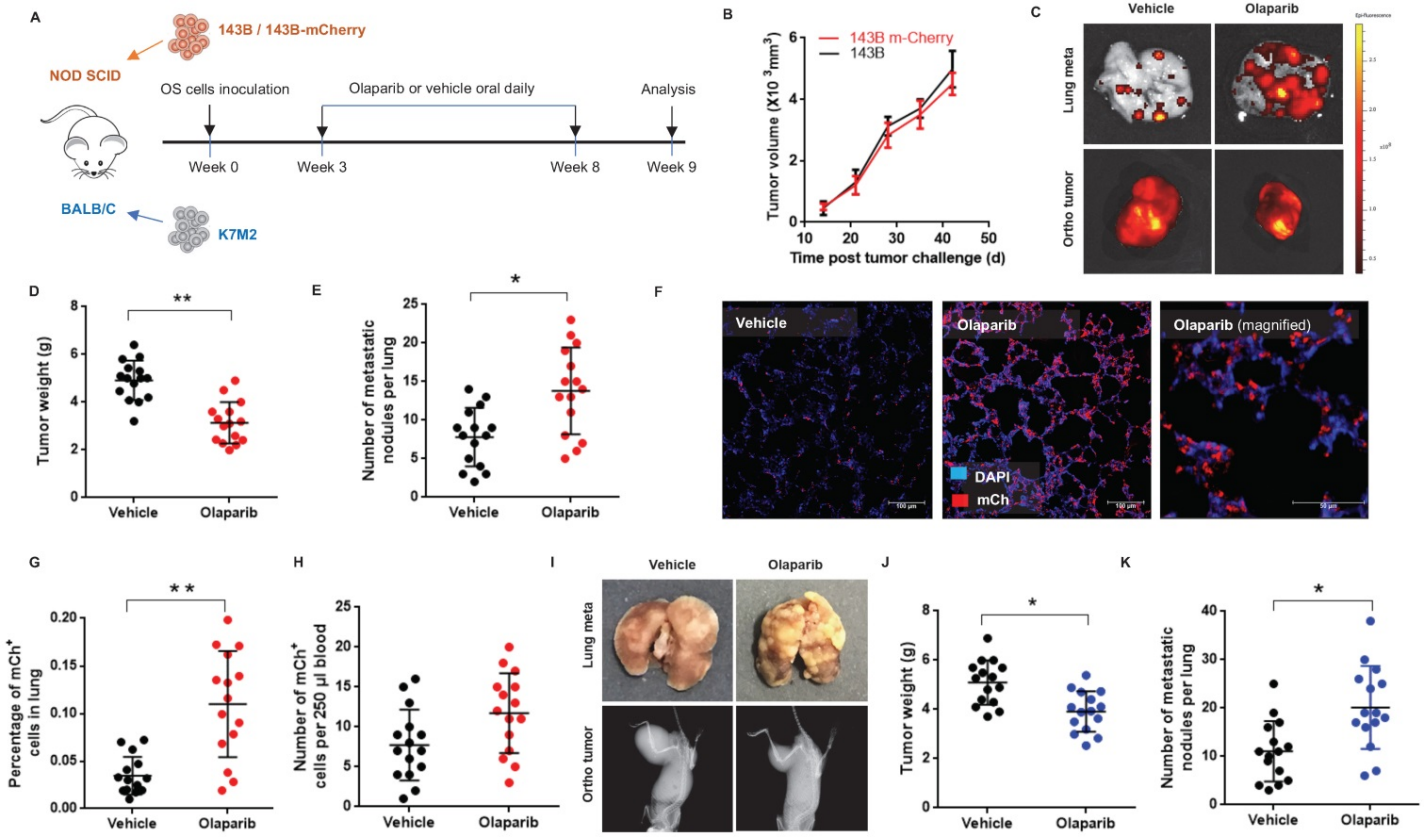
** Olaparib promoted pulmonary metastasis of osteosarcoma in tumor bearing mouse models. (A)** Drug scheduling in tumor bearing mice. **(B)** Volume of OS tissues from 143B or 143B-mCherry inoculated NOD/SCID mice, calculated by the formula: volume = (width)^2^ x length/2, with subtraction of normal tissue volume of the contraleteral non-tumor hindlimb. n = 15 per group.** (C)** Representative fluorescence images of lung metastasis and X-Ray images for orthotopic 143B-mCherry tumors of NOD/SCID mice treated with olaparib or vehicle.** (D)** Weight of orthotopic 143B-mCherry tumors in NOD/SCID mice treated with olaparib or vehicle (mean ± s.e.m.; n = 15 mice per group). **(E)** Number of metastatic nodules per lung section in NOD/SCID mice (mean ± s.e.m.).** (F)** Representative fluorescence images of sections of lung from 143B-mCherry inoculated NOD/SCID mice with or without olaparib treatment. Scale bars, 100 μm. **(G)** Percentage of mCherry cells in lung analyzed by flow cytometry (n = 15 mice per group). **(H)** CTCs in blood of NOD/SCID mice with or without olaparib treatment analyzed by flow cytometry (n = 15 mice per group).** (I)** Representative images showing orthotopic K7M2 tumors and lung tissues in BALB/c mice. **(J)** Weight of orthotopic K7M2 tumors in BALB/c mice treated with olaparib or vehicle (n = 15 mice per group). **(K)** Number of metastatic nodules per lung section in BALB/c mice (mean ± s.e.m.).

The first FDA approved PARP inhibitor olaparib was selected for the examination of its effects on osteosarcoma growth and lung metastasis in two most commonly used orthotopic OS mouse models: immunodeficient NOD/SCID mice challenged with a human OS cell line 143B, and immunocompetent BALB/c mice challenged with a mouse OS cell line K7M2 (**Fig. 1A**). To trace metastasis, the 143B cells were modified to express a fluorescent mCherry (143B-mCh) protein, and it was verified that this modification did not affect the tumorigenesis of 143B (**Fig. 1B**). 6-week old mice were orthotopically inoculated with OS cells and allowed to grow for another 3 weeks. Then the tumor bearing mice received daily intragastric administration of either 100mg/kg olaparib or vehicle for 5 weeks (**Fig. 1A**). Treatment with olaparib had considerable inhibitory effect on the growth of primary tumors in both mouse models (**Fig. 1C, D, I, J**). However, it resulted in increased pulmonary tumor metastasis in both mouse models. In particular, olaparib dramatically aggravated lung metastases, including increased incidence of spontaneous metastases in the lungs (**Fig. 1C, E, I, K**), and higher percentage of micro-metastatic cells in lung tissues (**Fig. 1F, G**), as well as more circulating tumor cells (CTCs) in blood (**Fig. 1H**) compared with vehicle. Taken together, these results suggest that olaparib may aggravate lung metastasis in osteosarcoma mouse models.

### PARPi enhanced migratory and invasive properties of OS cells *in vitro*

**Figure 2:**
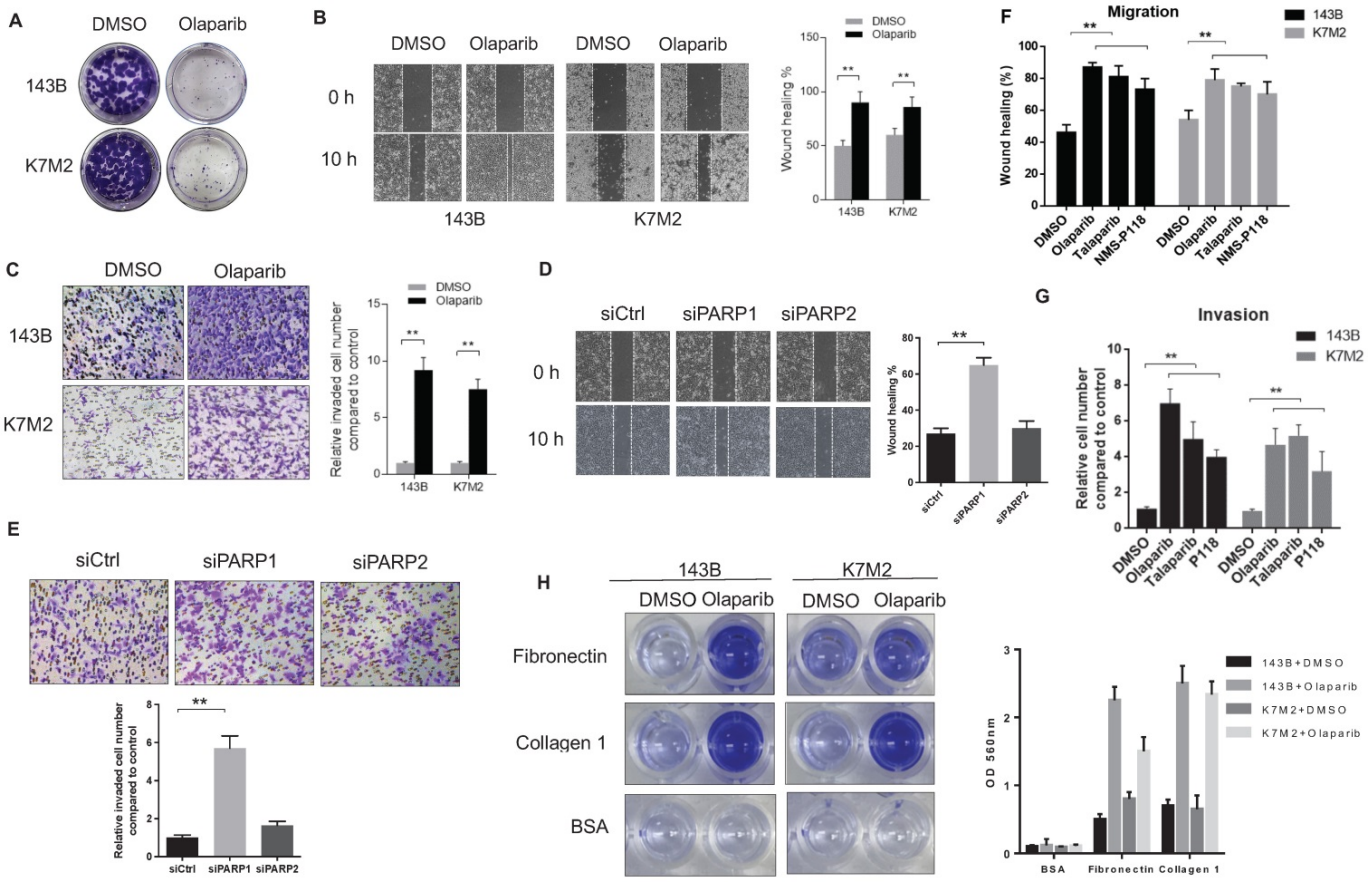
** The reduction of PARP1-mediated poly (ADP-ribose) enhanced the migratory and invasive properties of osteosarcoma cells. (A)** Colony formation assay for detection of the proliferation of 143B and K7M2 cells treated with DMSO or olaparib for 48 h.** (B)** Migratory properties of 143B and K7M2 cells treated with DMSO or olaparib were tested using wound healing assays. Quantification of percentage of wound closure was determined by analysis using ImageJ software. Mean +/-S.D. of three independent experiments. Scale bars, 300 μm. **(C)** Invasive behaviors in collagen-type-IV gels of 143B and K7M2 cells with DMSO or olaparib were observed in chamber invasion assay. Scale bars, 100 μm. **(D)** The effect of PARP1 and PARP2 knockdown on the migration properties of 143B cells was tested via wound healing assay. Scale bars, 300 μm.** (E)** The effect of PARP1 and PARP2 knockdown on the invasive behavior of 143B cells was tested by chamber invasion assay. Scale bars, 100 μm. **(F)** Wound healing assay for detection of the migrative property for OS cells treated with DMSO, PARP inhibitors, respectively. **(G)** Analysis of transwell invasion assay for OS cells treated with DMSO, PARP inhibitors, respectively. **(H)** Represented images of the adhesion ability of 143B and K7M2 cells treated with DMSO or olaparib on fibronectin or collagen-type-I coated plates. Quantification of the adherent cells was determined by the OD value at 560 nm.

To confirm the pro-metastatic role of PARPi in osteosarcoma that we observed in mouse models, we tested the activities of olaparib on the human OS cell line143B and the murine OS cell line (K7M2) *in vitro*. The colony formation assay revealed that olaparib exhibited good inhibitory effect on the proliferation of both OS cell lines (**Fig. 2A**). In cultured wound-healing assays, olaparib treated cells healed the wound much faster than untreated cells (**Fig. 2B**). In addition, after olaparib treatment, both OS cells could migrate through the collagen gel more efficiently in the chamber invasion assay (**Fig. 2C**). Interestingly, enhanced pro-migratory and pro-invasive effects were only observed in PARP1 knockdown 143B cells but not PARP2 knockdown cells compared with control group (**Fig. 2D, E**), suggesting the PARP1 dependence of this pro-metastatic activity. To verify whether olaparib promoted metastasis is a general effect on OS cells, we also examined the effect of two other PARP inhibitors (PARPi) - talaparib and NMS-P118 on the migration and invasion of two OS cell lines. As a result, increased migration and invasion in all PARPi treated cells were observed (**Fig. 2F, G**). More OS cell lines including U2OS and MG-63 were also tested for olaparib inhibition, consistently, faster wound healing and transwell ability were detected (**Supplementary Fig. S1**). In addition, the treatment of olaparib could also considerably enhance the speed of OS cell adhesions on different extracellular matrix (ECM) coated surface, including collagen I and fibronectin (**Fig. 2H, Supplementary Fig. S2**).

### PARP inhibition induced epithelial-mesenchymal transition in osteosarcoma

**Figure 3:**
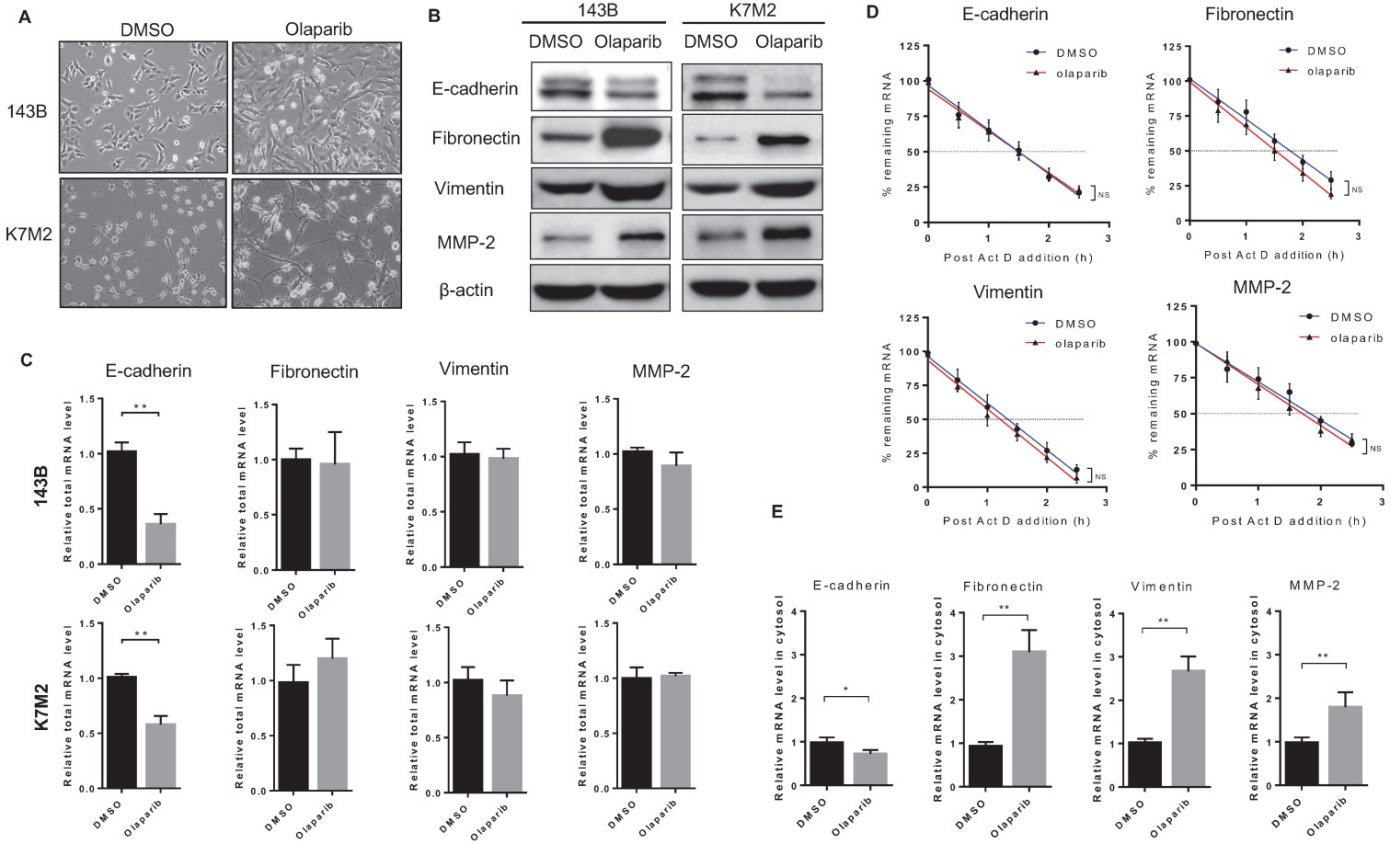
** PARPi treatment induced EMT of osteosarcoma cells. (A)** Phase-contrast images showing the EMT morphology change of DMSO and olaparib treated cells. Scale bars, 100 μm. **(B)** The effect of olaparib treatment on the protein level of EMT markers was determined by western blotting analysis. **(C)** The total RNA in OS cells treated with DMSO or olaparib was extracted and the effect of olaparib treatment on the mRNA level of EMT markers was determined by RT-qPCR. **(D)** DMSO or olaparib treated 143B cells were subjected to transcriptional inhibition with or without olaparib maintenance for various lengths of time as indicated. RT-qPCR was performed to assess the remaining mRNA levels of EMT markers. The half-lives of different samples are indicated in the inset. **(E)** The cytosol RNA in 143B cells treated with DMSO or olaparib was extracted and the effect of olaparib treatment on the mRNA level of EMT markers was determined by RT-qPCR.

In addition to increased migratory and invasive properties detected in OS cells after olaparib treatment, another phenomenon in morphology change for those cells was also observed. Compared to untreated cells, olaparib-treated cells appeared to adopt a more elongated and stretched shape (**Fig. 3A**), which showed resemblance to cells undergone epithelial-mesenchymal transition (EMT). In cancer types of mesenchymal origin like osteosarcoma, expressions of both epithelial and mesenchymal markers were found, and those cells could also undergo EMT process to a more metastatic state upon stimulations 19. To investigate if PARP inhibition led to EMT in OS cells, we examined the expressions of epithelial and mesenchymal markers in 143B and K7M2 cells with or without olaparib treatment. Western blot analysis revealed that the treatment of olaparib decreased the expression of the epithelial marker (E-cadherin) and increased the expression of the mesenchymal markers (vimentin, fibronectin and MMP-2) at translation level in both OS cell lines (**Fig. 3B**), suggesting that PARP inhibition could induce EMT in OS. Then we investigated the transcriptional changes of those markers induced by PARP1 inhibition in two OS cell lines using RT-qPCR. The results showed that olaparib decreased transcription of the epithelial gene *CDH1* encoding E-cadherin (**Fig. 3C**). Interestingly, no significant change in the mRNA level of mesenchymal marker genes (vimentin, fibronectin and MMP-2) after olaparib inhibition were observed (**Fig. 3C**). This contradiction suggests that the elevated mesenchymal marker proteins in olaparib treated OS cells were translated from limited mRNA transcripts, which were possibly to be more stabilized or more efficiently exported from the nucleus.

Therefore, we firstly looked at the mRNA half-time of the EMT markers in 143B cells with or without olaparib treatment. The half-time of four mRNAs did not vary significantly with or without olaparib treatment (**Fig. 3D**). When we quantified the mesenchymal marker mRNAs in cytosol fraction of cells treated with or without olaparib and detected the markers mRNA level dramatically increased in olaparib treated cells compared to the control cells (**Fig. 3E**). These suggest that the mRNA of those mesenchymal markers could be more efficiently exported from nucleus to cytosol via an olaparib-triggered mechanism, so that an elevated amount of protein translation for those genes could be achieved even when both half-time and the number of templates of those mRNAs were basically unchanged.

### Ezrin was identified as an interactive protein for PARP1

**Figure 4:**
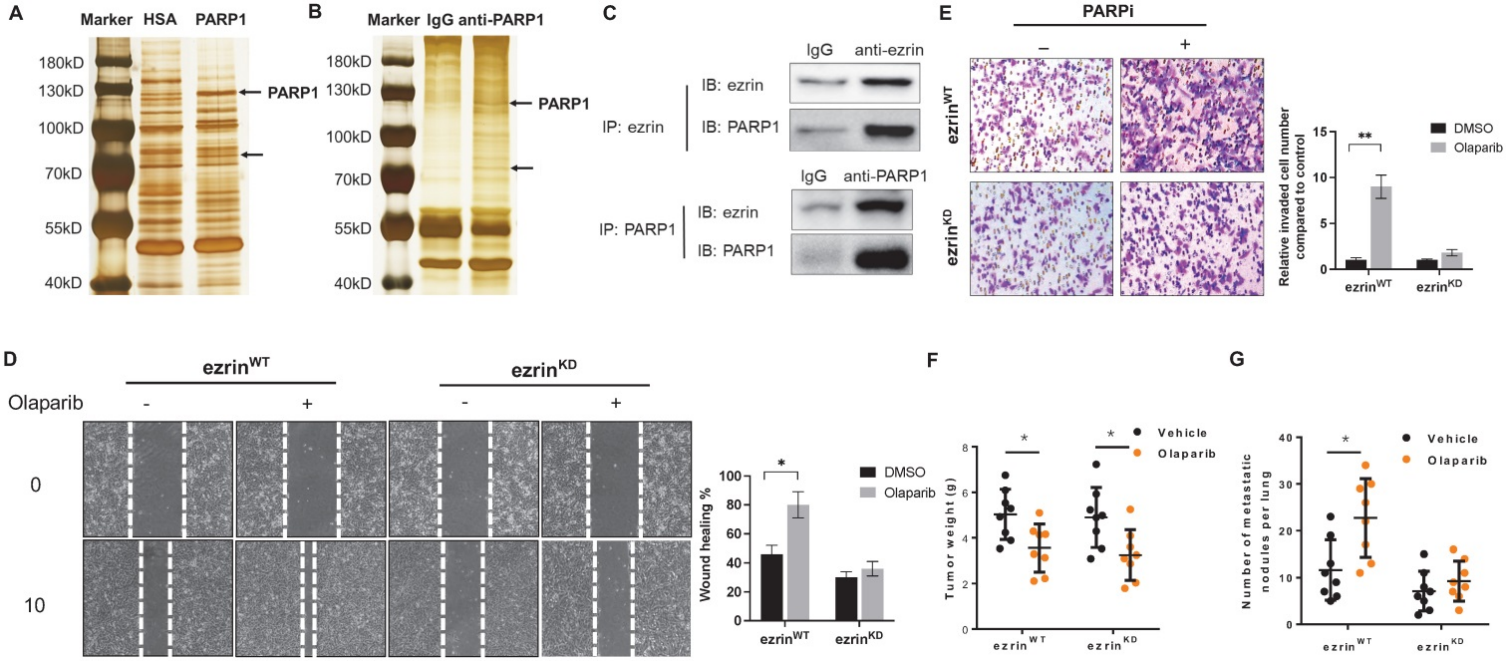
** Olaparib induced OS migration and invasion were ezrin-dependent. (A)** Human recombinant his-tagged PARP1 protein based pull-down assay was performed in OS cell lysates to identify PAPR1 interactive proteins. Human serum albumin (HSA) was used as control. The obtained proteins were separated in 10% SDS-PAGE gel flowed by silver staining. **(B)** co-IP assay for baiting PARP1 interactive proteins using anti-PARP1 antibody in OS cell lysates. IgG was used as negative control. The obtained proteins were separated in 10% SDS-PAGE gel flowed by silver staining. **(C)** 143B cell lysate was subjected for co-IP with anti-PARP1 or control IgG antibodies followed by western blotting with the indicated antibodies to verify the interaction between ezrin and PARP1 (n = 3). **(D)** Representative images from wound healing assay showing the effect of olaparib on cell migration of ezrin wildtype (ezrin^WT^) and ezrin knock down (ezrin^KD^) 143B cells. Wound spaces were analyzed using ImageJ. Scale bars, 300 μm. **(E)** Representative images from transwell assay showing the effect of olaparib on cell invasion of ezrin wildtype (ezrin^WT^) and ezrin knock down (ezrin^KD^) 143B cells. Invaded cells were counted in 15 random fields on the lower surface of the filters and expressed as ratio (fold) of invaded cells compared with the vehicle group. Scale bars, 300 μm.** (F)** Weight of NOD/SCID mice bearing orthotopic ezrin^WT^ or ezrin^KD^ 143B-mCherry tumors treated with olaparib or vehicle (mean ± s.e.m.; n = 8 mice per group). The statistical analyses were performed using one-way ANOVA with Tukey's multiple comparison tests. **(G)** Number of metastatic nodules per lung in NOD/SCID mice treated with olaparib or vehicle.

To further explore the mechanism of PARP1 inhibition promoted metastasis in OS, tracing the critical interactive proteins for PARP1 in OS cells is a good approach. We performed a pull-down assay using human recombinant PARP1 protein as the bait in cell lysate of 143B cells, and human serum albumin (HSA) was used as a negative control (**Fig. 4A**). A co-IP assay using an anti-PARP1 antibody was also conducted to eliminate the non-specific binding proteins (**Fig. 4B**). As a result, a distinctive band at approximately 80kDa compared with control was observed in both gels after silver staining (**Fig. 4A, B**). Analysis of proteins in the indicated band from each gel by mass spectrometry provided a candidate list of possible interactive proteins (**Supplementary Table 3**). There were two candidates appeared in both lists: ezrin and cortactin. Interactions of the two proteins with PARP1 were verified by co-IP assays. Ezrin, a protein reported closely associated with osteosarcoma metastasis, was confirmed to be an interactive protein for PARP1 (**Fig. 4C**). However, the interaction of another protein cortactin with PARP1 was not detected (**Supplementary Fig. S3**).

Ezrin belongs to the ERM (ezrin/radixin/moesin) family, which organize complex membrane domains by interacting with plasma membrane proteins, phospholipids and the cytoskeleton 22. To verify whether ezrin was associated with PARPi induced OS metastasis, 143B cells were subjected to lentivirus-mediated, shRNA-directed stable knockdown of ezrin (ezrin^KD^). Compared with the ezrin wildtype 143B cells (ezrin^WT^), the olaparib induced migration and invasion were significantly decreased in ezrin^KD^ cells *in vitro* (**Fig. 4E, D**). In OS mouse models with inoculation of ezrin^KD^ and ezrin^WT^ 143B cells in NOD/SCID mice, the tumor weight of two groups of mice were comparable, and the reduction in tumor size after olaparib treatment did not show significant difference (**Fig. 4F**). However, the number of lung metastatic nodules in ezrin^KD^ group was dramatically lower than ezrin^WT^ group, and olaparib aggravated lung metastasis in ezrin^WT^ group was considerably relieved in ezrin^KD^ group (**Fig. 4G).**

### PARP1 regulated the phosphorylation of ezrin

**Figure 5:**
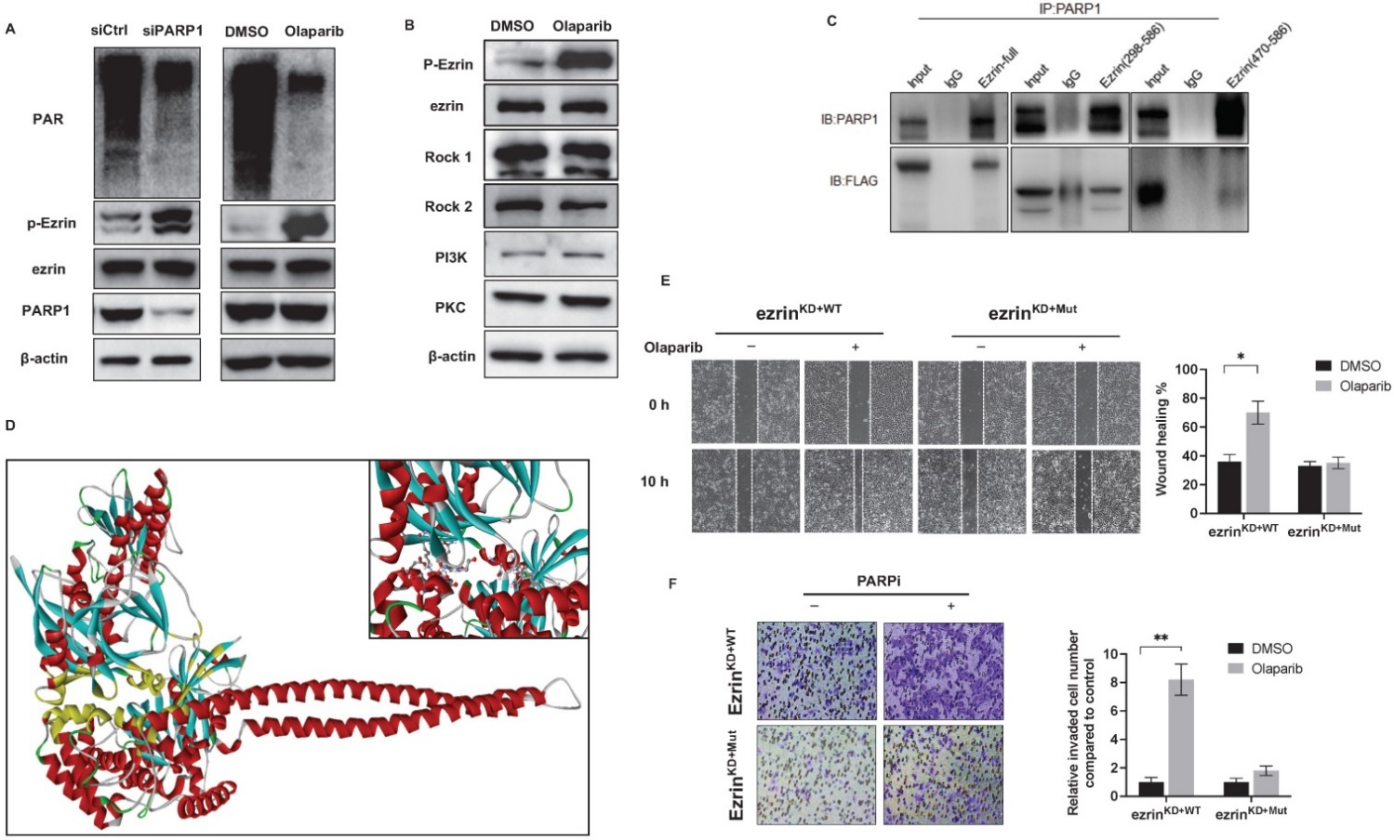
** The PARylation of ezrin triggered by PARP1 hindered its phosphorylation. (A)** siRNA induced PARP1 knock down and PARP enzymatic inhibition with olaparib both increased ezrin phosphorylation without affecting the total ezrin. The protein level of PAR, p-Ezrin, ezrin and PARP1 was determined using western blotting. β-actin was used as a loading control.** (B)** Olaparib promoted the phosphorylation of p-Ezrin independent on the kinases. **(C)** Binding domains of ezrin with PARP1 identified by co-IP using truncated versions of ezrin was verified by western blotting.** (D)** Molecular modeling of the interaction between ezrin and PARP1 interaction at full length. **(E)** Representative images from wound healing assay showing the effect of olaparib on cell migration of ezrin^KD+WT^ and ezrin^KD+Mut^ 143B cells. Wound spaces were analyzed using ImageJ. Scale bars, 300 μm. **(F)** Representative images from transwell assay showing the effect of olaparib on cell invasion of ezrin^KD+WT^ and ezrin^KD+Mut^ 143B cells. Invaded cells were counted in 15 random fields on the lower surface of the filters and expressed as ratio (fold) of invaded cells compared with the vehicle group.

The activity of ezrin mainly relies on its phosphorylation in its C-terminal as an active form. The phosphorylated ezrin (p-Ezrin) could interact with F-actin, and subsequently regulates cell motility, polarity and adherence which are closely related to cancer metastasis 22. The interaction between PARP1 and ezrin, suggesting that the interaction domain could affect the activation of ezrin. We found that either knockdown PARP1 by shRNA or enzymatic inhibition of PARP1 by olaparib significantly elevated the phosphorylation level of ezrin without affecting the expression of ezrin (**Fig. 5A**). To further confirm the olaparib induced phosphorylation of ezrin was dependent on PARP1 interaction but not its phosphorylation kinases, the levels of the kinase were examined, with no significant change with or without olaparib treatment (**Fig. 5B**).

To provide structural evidence for PARP1 and ezrin interaction, and its effect on ezrin phosphorylation, the interactive domain of ezrin with PARP1 was determined by immunoprecipitation in 143B cells transfected with flag-labeled truncated versions of ezrin containing different domains (ezrin: full length 1-586aa, 298-586aa, 470-586aa). The results showed that PARP1 bound with the ERMAD domain (298-470aa) of ezrin (**Fig. 5C**), which is the same domain with phosphorylation. A molecular docking of PARP1-ezrin interaction was conducted representing the binding domain and site (**Fig. 5D**), and the C-ERMAD domain of ezrin was involved in the interaction with PARP1. In ezrin^KD^ 143B cells, we transfected the cells with a plasmid either expressing wildtype ezrin (ezrin^KD+WT^) or an ezrin mutant on T567 of the phosphorylation site (ezrin^KD+Mut^), which could not be phosphorylated. We observed that olaparib induced migration and invasion was considerably reduced in ezrin^KD+WT^ cells than ezrin^KD+Mut^ cells (**Fig. 5E, F**), suggesting the PARPi induced OS metastasis could be closely related to ezrin phosphorylation and associated functions.

### Phosphorylated ezrin localized in the nuclear speckle and promoted RNA export

**Figure 6:**
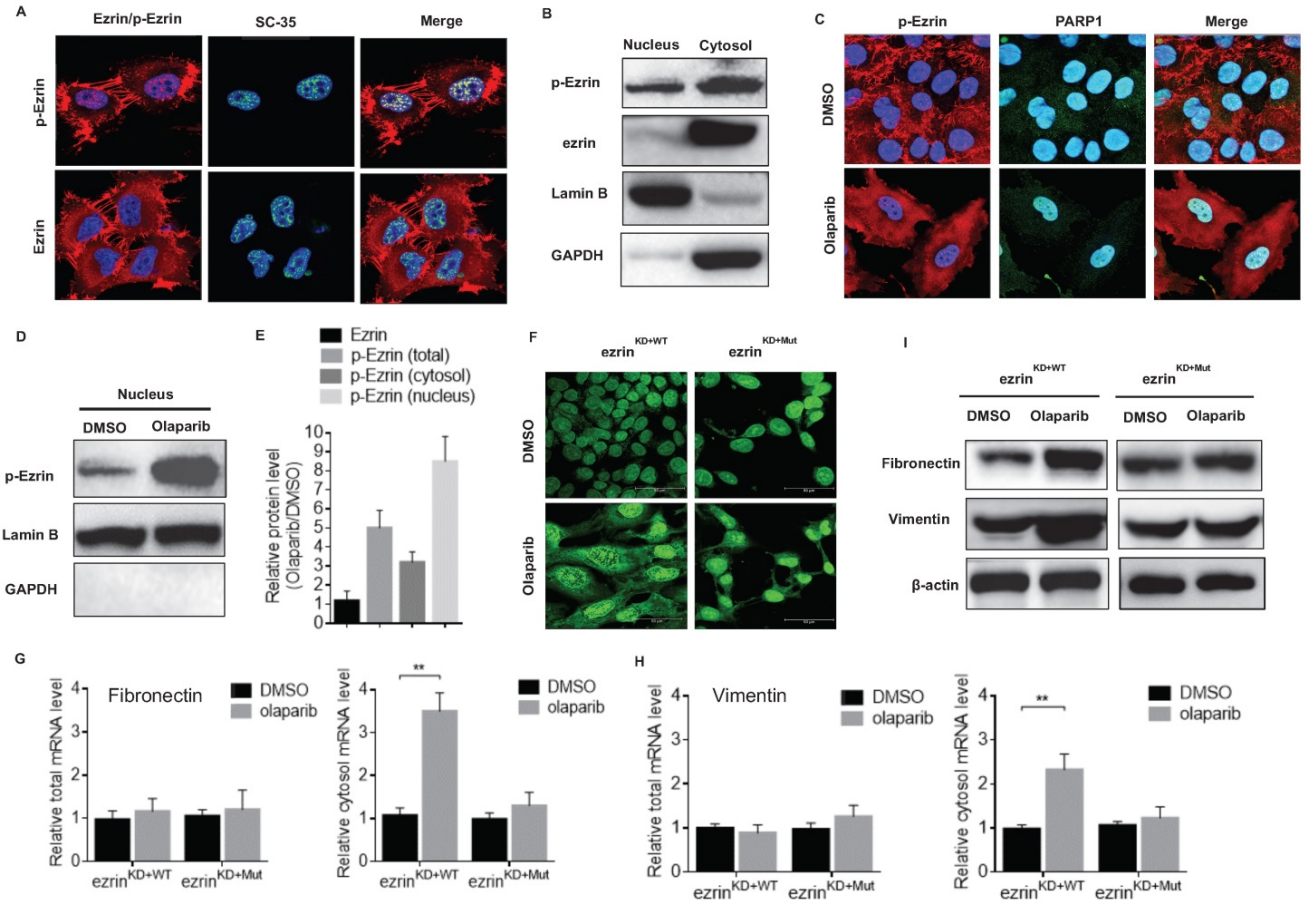
** Phosphorylated ezrin localized in the nucleus speckle and promoted RNA export. (A)** The subcellular localization of p-Ezrin and ezrin in 143B cells and their co-localization with SC-35, a nuclear speckle marker.** (B)** Western blot analysis for the localization of p-Ezrin and ezrin in different cellular components in OS cells. Detection of Lamin B was used as a loading control.** (C)** Confocal imaging of p-Ezrin level in nucleus in OS cells treated with or without olaparib treatment. **(D)** The level of p-Ezrin in the nuclear proteins isolated from OS cells treated with or without olaparib determined by western blotting. **(E)** The relative protein level of ezrin, p-Ezrin in olaparib treated cells compared with the vehicle was calculated according to the western blotting analysis using ImageJ software. The western blotting was repeated at least three times. **(F)** Representative immunofluorescence images of RNA export in ezrin^KD+WT^ or ezrin^KD+Mut^ cells treated with DMSO, olaparib. Scale bar = 50 μm. **(G)** The mRNA of fibronectin in the cytosol or the whole ezrin^KO+WT^ or ezrin^KO+Mut^ cells treated with DMSO or olaparib. **(H)** The mRNA of vimentin in the cytosol or the whole ezrin^KD+WT^ or ezrin^KD+Mut^ cells treated with DMSO or olaparib. **(I)** Western blotting analysis of the protein level of fibronectin and vimentin in ezrin^KD+WT^ or ezrin^KD+Mut^ cells treated with DMSO or olaparib. β-actin was used as a loading control.

Noteworthy, an interesting nucleus localization of p-Ezrin in 143B cells was observed from the confocal microscopy images (**Fig. 6A**). The nucleus localization of p-Ezrin was distributed as foci and the pattern is very similar to that of nuclear speckle 38. Therefore, we checked the co-localization of p-Ezrin with the nuclear speckle marker SC-35, and they perfectly co-localized with each other (**Fig. 6A**), indicating the nuclear speckle localization of ezrin. A relatively high proportion of p-Ezrin was also detected in the nucleus fraction of cell components compared to total ezrin by western blot (**Fig. 6B**). After olaparib treatment, p-Ezrin at cell membrane was considerably decreased, more dispersed distribution of p-Ezrin in the cytoplasm was observed in confocal images (**Fig. 6C**). Importantly, olaparib treatment not only increased the total p-Ezrin level, it also significantly promoted the distribution of p-Ezrin in the nucleus in nuclear speckle (**Fig. 6C, D, E**). Nuclear speckle is a cellular structure closely associated with RNA processing and export 38. Considering the enhanced translation efficiency of EMT marker mRNAs mentioned above, it is highly possible the nuclear speckle localization of p-Ezrin was related to mRNA nucleus export. Therefore, we examined the effect of olaparib on RNA distribution in ezrin^KD+WT^ or ezrin^KD+Mut^ 143B cells, and we found that olaparib treatment increased the distribution of RNA in cytoplasm in ezrin^KD+WT^ cells, however the lack of ezrin phosphorylation in ezrin^KD+Mut^ cells exhibited a higher RNA accumulation in nucleus (**Fig. 6F**). Then we checked the total or cytosol mRNA level of fibronectin and vimentin in ezrin^KD+WT^ or ezrin^KD+Mut^ 143B cells. For both marker genes, the total mRNAs remained the same, and an elevated mRNA level in cytosol was observed in olaparib treated ezrin^KD+WT^ cells, while no observable increase in mRNA level in cytosol was found in ezrin^KD+Mut^ cells (**Fig. 6G, H**). At protein translation level, olaparib induced rise in fibronectin and vimentin was also alleviated when lacking ezrin phosphorylation in ezrin^KD+Mut^ cells (**Fig. 6I**). All these suggest that the elevated amount of p-Ezrin in nucleus speckle accelerated the export of mRNAs of mesenchymal marker genes, promoting the translation of those marker proteins without affecting the mRNA at transcription level.

### Ezrin inhibitor rescued olaparib induced OS lung metastasis *in vivo*

The above data suggested that the PARPi induced OS metastasis could be closely related to ezrin phosphorylation. Therefore, during Olaparib treatment, inhibition of ezrin activation using an ezrin inhibitor NSC668394 (EZRi) might rescue olaparib induced metastasis. We evaluated the anti-proliferation, migration and invasion ability of EZRi, PARP inhibitor, EZRi combined with PARP inhibitor in OS cells. The EZRi significantly inhibited the phosphorylation of ezrin without affecting the expression of ezrin (**Supplementary Fig. S4A**). The combination of EZRi and olaparib had comparable activity on cell proliferation ability with olaparib only (**Supplementary Fig. S4B**). Notably, the combination of EZRi with olaparib dramatically hindered cell migration and invasion (**Supplementary Fig. S4C, D**).

**Figure 7:**
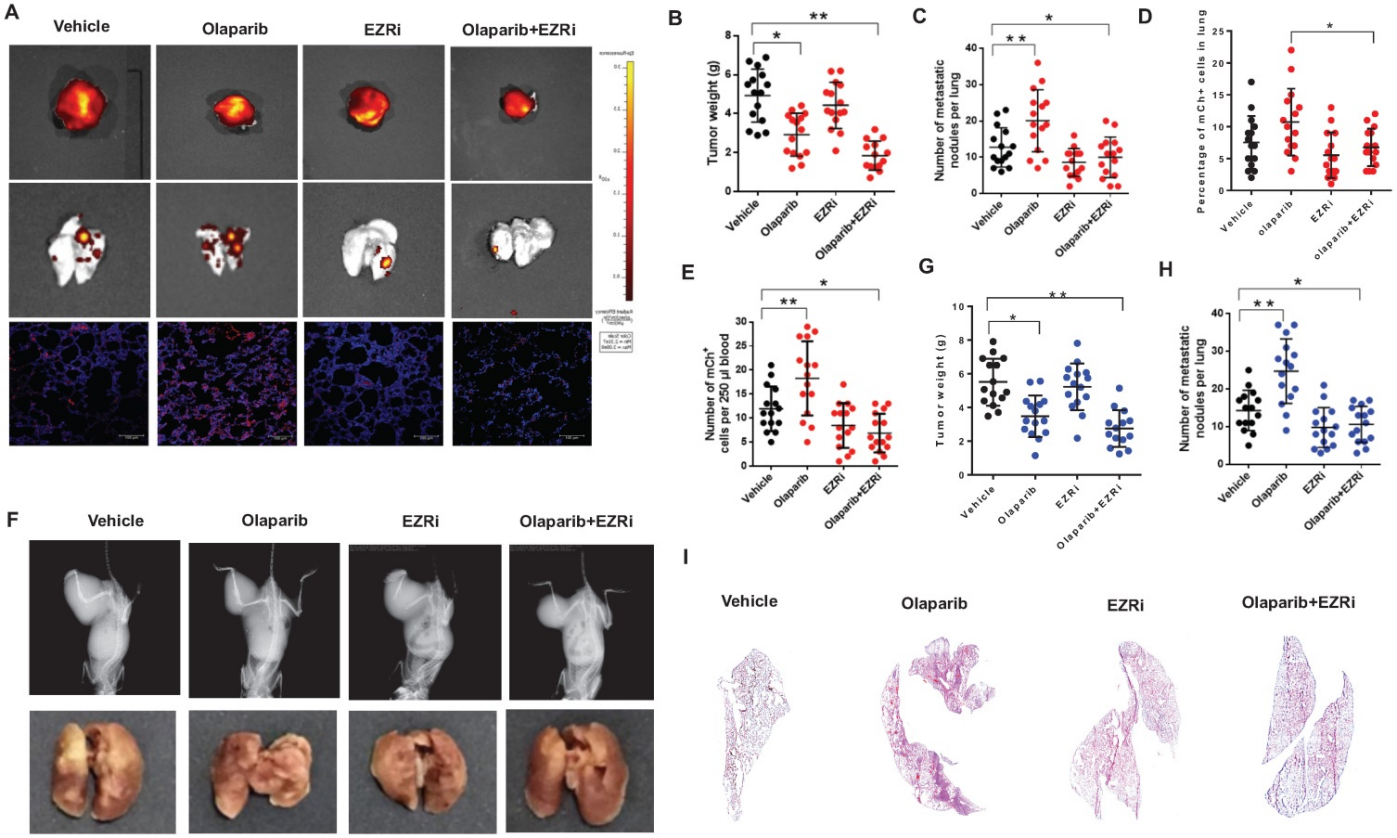
** The combination of olaparib with an ezrin inhibitor NSC668394 (EZRi) rescued olaparib induced OS lung metastasis. (A)** Representative biophonic images of OS tumor, lung metastasis, and representative images of mCherry fluorescence of micro-metastasis in lung sections in 143B-mCherry inoculated NOD/SCID mice treated with vehicle, Olaparib, EZRi or the combination of Olaparib and EZRi.** (B)** Weight of orthotopic 143B-mCherry tumors in 143B-mCherry inoculated NOD/SCID mice treated with vehicle, olaparib, EZRi or the combination of olaparib and EZRi (n = 15 mice per group). **(C)** Number of metastatic 143B-mCherry nodules visible in lung in 143B-mCherry inoculated NOD/SCID mice with each treatment (mean ± s.e.m.).** (D)** Percentage of 143B-mCh^+^ in lung of 143B-mCherry inoculated NOD/SCID mice with each treatment analyzed by flow cytometry (n = 15 mice per group).** (E)** Number of 143B-mCherry CTCs in blood of 143B-mCherry inoculated NOD/SCID mice with each treatment analyzed by flow cytometry (n = 15 mice per group).** (F)** Representative images of OS tumor and lung metastasis in K7M2 inoculated BALB/c mice treated with vehicle, olaparib, EZRi or the combination of olaparib and EZRi. **(G)** Weight of orthotopic K7M2 tumors in BALB/c mice treated with vehicle, olaparib, EZRi or the combination of olaparib and EZRi (n = 15 mice per group). **(H)** Number of metastatic K7M2 nodules visible in lung in K7M2 inoculated BALB/c mice with each treatment.** (I)** Representative H&E images of lung sections after each treatment showing the metastatic tumors.

Two mouse models described above were used to investigate the effects on tumorigenesis after the treatment of vehicle, olaparib, EZRi, the combination of olaparib and EZRi. The tumor size, visible lung metastasis and fluorescent micro-metastasis in lung sections were analyzed and represented images of 143B-SCID mice were shown (**Fig. 7A**). Ezrin inhibitor combined with olaparib had significant inhibitory activity on the growth of primary tumors in NOD/SCID mice (**Fig. 7A-E**). In addition, ezrin inhibition could dramatically decreased lung metastases, including decreased the incidence (**Fig. 7A-C**), lower percentage of micro-metastatic cells in lung tissues (**Fig. 7D**), as well as less circulating tumor cells (CTCs) in blood (**Fig. 7E**) compared with vehicle. In K7M2- BALB/c mouse model, decreased tumor growth and lung metastasis were also observed for olaparib and ezrin combinational group (**Fig. 7F-I**). Taken together, these results indicate that ezrin inhibition could reduce the lung metastasis induced by PARP inhibitor olaparib.

## Discussion

In this work, using two OS orthotopic mouse models, we discovered that although PARPi exhibited significant inhibitory effects on the growth of the orthotopic OS tumors, unexpectedly, the treatment of PARPi essentially aggravated the pulmonary metastasis and led to decreased overall survivals. We found that PARP inhibition induced EMT and dramatically increased the expression of mesenchymal markers in OS cells. Furthermore, we discovered that PARP1 interacted with ezrin, and the PARP inhibition boosted the phosphorylation of ezrin. Besides the traditional knowledge on the functions of p-Ezrin on cell membrane, we newly discovered its nuclear speckle localization and function in mRNA export. PARP inhibition elevated the accumulation of p-Ezrin in nuclear speckle and accelerated the export of EMT related gene mRNAs. The combination of PARP inhibitor with ezrin phosphorylation inhibitor effectively reduced both tumor growth and lung metastasis in OS mouse models.

PARP inhibitors have shown great potential in clinical treatment of breast and ovarian cancer. The pro-metastatic roles of PARP1 in a range of cancer types were well-established 39, 40. However, the pro-metastatic effect of PARP inhibitors was also reported by other teams. In a study conducted by Zuo et al, olaparib was also found promoting breast cancer bone metastasis through PARP2, not PARP1 specifically in the myeloid lineage 41. Additionally, in terms of sarcoma, a phase II study of olaparib in adults with recurrent/metastatic Ewing's sarcoma showed no significant responses or durable disease control 42. Another phase II study of talazoparib in patients with advanced or recurrent solid tumors including sarcoma showed no significant improvement in overall survival under talazoparib treatment. As one type of sarcoma, osteosarcoma shares similar characterizations and it could be possible that PARPi induced ezrin phosphorylation and OS metastasis discovered in this study is related with the failure of these clinical trials. Besides, it could also be possible that PARP1-Ezrin axis is relatively more dominant in osteosarcoma compared to other signaling pathways associated with cell migration and metastasis.

The roles of PARP1 in EMT in cancer have been investigated in a range of cancer types and the effects of PARPi also remain controversial. In a murine model of metastatic melanoma, PARP inhibition counteracted the ability of melanoma cells to metastasize to the lung 43. While in a PARP1 knockout transgenic prostate adenocarcinoma mouse model, impaired PARP-1 function increased levels of transforming growth factor-β (TGF-β) and promoted prostate tumorigenesis *in vivo* via TGF-β-induced EMT 44. Mechanism studies showed PARP-1 dissociates Smad complexes from DNA by ADP-ribosylation, which attenuates Smad-specific gene responses and TGF-β-induced epithelial-mesenchymal transition 45. Furthermore, PARP1 was also found bound to FN1 promoter together with Snail1 and the p65 subunit of NF-κB and regulated mesenchymal gene transcription 46. In OS cells of mesenchymal origin, we found PARP1 inhibition could also induce EMT process mainly by elevating the translation of mesenchymal marker genes without affecting the transcription stage. PARPi increased the accumulation of p-Ezrin in nuclear speckles, boosted mRNA nuclear export and accelerated the subsequent translation of mRNAs for mesenchymal marker genes, which aggravated EMT process.

Since the first identification of ezrin in metastatic pediatric sarcoma by Khanna and co-workers, collective data have provided compelling evidence for a metastasis-promoting function of ezrin in osteosarcoma 20, 21, 47. Here we discovered that phosphorylation of ezrin could be regulated by PARP1, possibly by the steric hindrance due to the same domain for PARP1 interaction and phosphorylation. More importantly, the localization and functions of ezrin were mainly reported in cytoplasm or taken place at plasma membrane, we firstly discovered the localization of p-Ezrin in nuclear speckle and revealed its function in mRNA export, which enriched the biological understanding for ezrin.

In summary, our investigation on the effect of PARPi on OS unveiled a novel mechanism for PARPi induced OS metastasis. The inhibition of PARP1 enzymatic activity aggravated EMT induction and activated ezrin by boosting phosphorylation. The pro-metastasis role of PARP inhibitors should be carefully considered in clinical treatment especially in tumors of sarcoma-origin. Further study is necessary to explore the clinical potential of combination therapy of PARP inhibitor with ezrin inhibitor in OS treatment.

## Supplementary Material

Supplementary figures and tables.

## Abbreviations

ATCC: American Type Culture Collection
ECM: Extracellular matrix
EMT: Epithelial-mesenchymal transition
IP: immunoprecipitation
KD: Knock down
OS: osteosarcoma
PARP: Poly (ADP-ribose) polymerase
PARPi: poly (ADP-ribose) polymerase inhibitors
PAR: Poly ADP-ribose
qRT-PCR: quantitative real-time reverse transcription-polymerase chain reaction
TFs: Transcription factors
WT: Wild-type
