# Supplementary material for: Poly (ADP-ribose) polymerase 1 (PARP1) inhibition promotes pulmonary metastasis of osteosarcoma by boosting ezrin phosphorylation
Source: Int J Biol Sci. 2022 Jan 9;18(3):1238–53. doi: 10.7150/ijbs.58784 (PMC8771856; doi:10.7150/ijbs.58784)
Supplement: Supplementary file 1 — Supplementary figures and tables. [file ijbsv18p1238s1.pdf]

S Table 1. The sequences of primers for EMT markers mRNA amplification in RT-qPCR assays.

Gene	Forward primer	Reverse primer
E-cadherin	5’-CAGCACGTACACAGCCCTAA-3’	5’-ACCTGAGGCTTTGGATTCCT-3’
Fibronectin	5’-CCGTGGGCAACTCTGTC-3’	5’-TGCGGCAGTTGTCACAG-3’
Vimentin	5′-CCTGCAATCTTTCAGACAGG-3′	5′-CTCCTGGATTTCCTCTTCGT-3′
MMP2	5′-GTCTTCCCCTTCACTTTTCTG-3’	5′-CGGAAGTTCTTGGTGTAGGTG-3’
β-actin	5’-TCCCTGGAGAAGAGCTACGA-3’	5’-AGCACTGTGTTGGCGTACAG-3’
GAPDH	5′-CATGACCACAGTCCATGCCAT-3′	5′-AAGGCCATGCCAGTGAGCTTC-3′

S Table 2. siRNA sequences used in transfection assay.

Target gene	siRNA sequence
ezrin	5’-GUGGGAUGCUCAAAGAUAAdTdT-3’
PARP1	5’-GGGCAAGCACAGUGUCAAAdTdT-3’
PARP2	5’-GGAGAAGGAUGGUGAGAAAdTdT-3’
Negative control	5’-UUCUCCGAACGUGUCACGUdTdT-3’

S Table 3. Lists of candidate proteins from the indicated bands in pull-down and co-IP assays analyzed by Mass spectrometry. Overlapped proteins from three assays were highlighted in red (ezrin) and green (cortactin).

	PARP1 pull down	PARP1 co-IP
1	**Ezrin OS=Homo sapiens OX=9606 GN=EZR PE=1 SV=4**	DNA-(apurinic or apyrimidinic site) lyase OS=Homo sapiens OX=9606 GN=APEX1 PE=1 SV=2
2	Beta-adrenergic receptor kinase 1 OS=Homo sapiens OX=9606 GN=GRK2 PE=1 SV=2	Nucleophosmin OS=Homo sapiens OX=9606 GN=NPM1 PE=1 SV=2
3	**Src substrate cortactin OS=Homo sapiens OX=9606 GN=CTTN PE=1 SV=2**	**Ezrin OS=Homo sapiens OX=9606 GN=EZR PE=1 SV=4**
4	Heterogeneous nuclear ribonucleoproteins A2/B1 OS=Homo sapiens OX=9606 GN=HNRNPA2B1 PE=1 SV=2	Casein kinase I isoform alpha OS=Homo sapiens OX=9606 GN=CSNK1A1 PE=1 SV=2
5	X-ray repair cross-complementing protein 5 OS=Homo sapiens OX=9606 GN=XRCC5 PE=1 SV=3	Glyceraldehyde-3-phosphate dehydrogenase OS=Homo sapiens OX=9606 GN=GAPDH PE=1 SV=3
6	Filaggrin-2 OS=Homo sapiens OX=9606 GN=FLG2 PE=1 SV=1	Annexin A2 OS=Homo sapiens OX=9606 GN=ANXA2 PE=1 SV=2
7	Putative elongation factor 1-alpha-like 3 OS=Homo sapiens OX=9606 GN=EEF1A1P5 PE=5 SV=1	Bifunctional methylenetetrahydrofolate dehydrogenase/cyclohydrolase, mitochondrial OS=Homo sapiens OX=9606 GN=MTHFD2 PE=1 SV=2
8	WD repeat-containing protein 70 OS=Homo sapiens OX=9606 GN=WDR70 PE=1 SV=1	**Src substrate cortactin OS=Homo sapiens OX=9606 GN=CTTN PE=1 SV=2**
9	Mitochondrial proton/calcium exchanger protein OS=Homo sapiens OX=9606 GN=LETM1 PE=1 SV=1	Eukaryotic translation initiation factor 2 subunit 1 OS=Homo sapiens OX=9606 GN=EIF2S1 PE=1 SV=3
10	Ataxin-2-like protein OS=Homo sapiens OX=9606 GN=ATXN2L PE=1 SV=2	Leukocyte receptor cluster member 1 OS=Homo sapiens OX=9606 GN=LENG1 PE=1 SV=1
11	Desmoplakin OS=Homo sapiens OX=9606 GN=DSP PE=1 SV=3	Tryptophan--tRNA ligase, mitochondrial OS=Homo sapiens OX=9606 GN=WARS2 PE=1 SV=1
12	Stromal interaction molecule 1 OS=Homo sapiens OX=9606 GN=STIM1 PE=1 SV=3	UPF0688 protein C1orf174 OS=Homo sapiens OX=9606 GN=C1orf174 PE=1 SV=2
13	Junction plakoglobin OS=Homo sapiens OX=9606 GN=JUP PE=1 SV=3	
14	RNA-binding protein EWS OS=Homo sapiens OX=9606 GN=EWSR1 PE=1 SV=1	
15	Anillin OS=Homo sapiens OX=9606 GN=ANLN PE=1 SV=2	
16	Ribonucleoprotein PTB-binding 1 OS=Homo sapiens OX=9606 GN=RAVER1 PE=1 SV=1	
17	Putative elongation factor 1-alpha-like 3 OS=Homo sapiens OX=9606 GN=EEF1A1P5 PE=5 SV=1	
18	SLAIN motif-containing protein 2 OS=Homo sapiens OX=9606 GN=SLAIN2 PE=1 SV=2	

Figure S1.

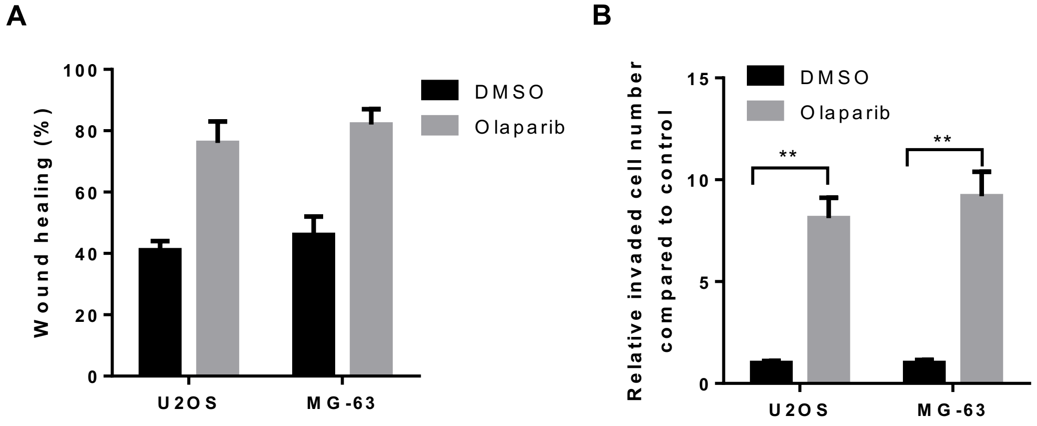

Supplementary Fig 1. Olaparib enhances the migratory and invasive functions of osteosarcoma cells *in vitro*. (A) Migratory properties of U2OS and MG-63 cells treated with DMSO or olaparib were tested using wound healing assays. Results showed that olaparib treated OS cells migrated faster compared to the control group. **(B)** Invasive behaviors in collagen-type-IV gels of U2OS and MG-63 cells with DMSO or olaparib were observed in chamber invasion assay. The relative invaded cells compared to control was counted by statistical. Results showed that a higher number of olaparib treated OS cells could invade through the membrane compared to the control group.

Figure S2.

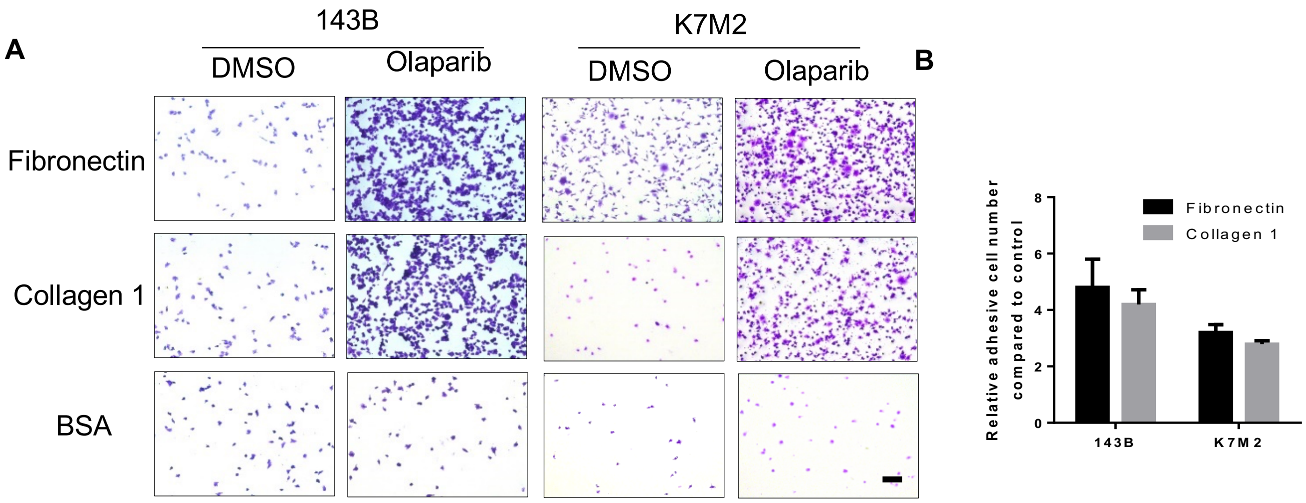

Supplementary Fig 2. Olaparib enhances the adhesive property of osteosarcoma cells *in vitro*. Representative diagrams for stained cells. DMSO or olaparib treated OS cells were incubated with BSA, fibronectin or collagen I coated wells for 1 h. Adherent cells were fixed and stained. **(A)** Representative diagrams for stained cells. Scale bar = 50 μm. **(B)** Quantification of the adherent cells.

Figure S3.

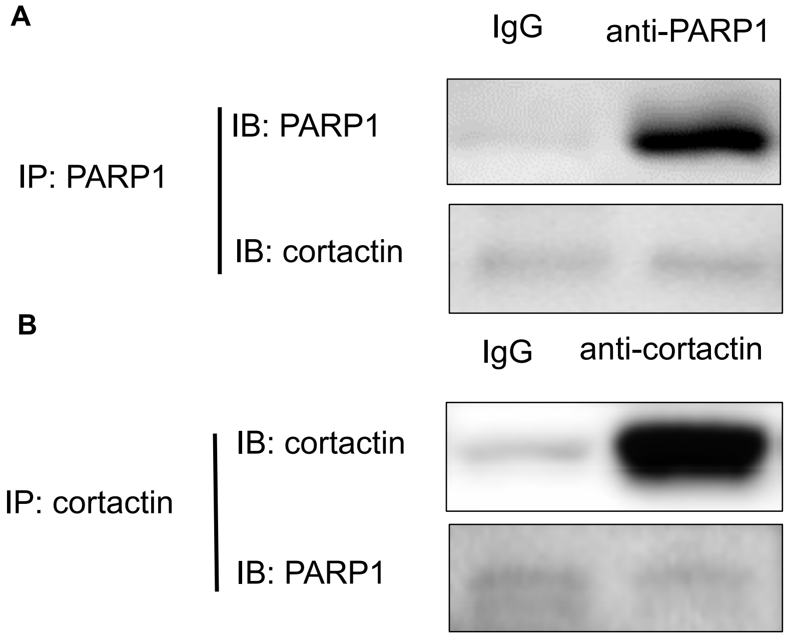

Supplementary Fig 3. PARP1 has no interaction with cortactin. 143B cell lysate was subjected for co-IP with anti-PARP1, anti-cortactin, or control IgG antibodies followed by western blotting with the indicated antibodies. **(A)** PARP1 antibody could not pull down cortactin protein. **(B)** Cortactin antibody could not pull down PARP1 protein. There was no interaction between PARP1 and cortactin.

Figure S4.

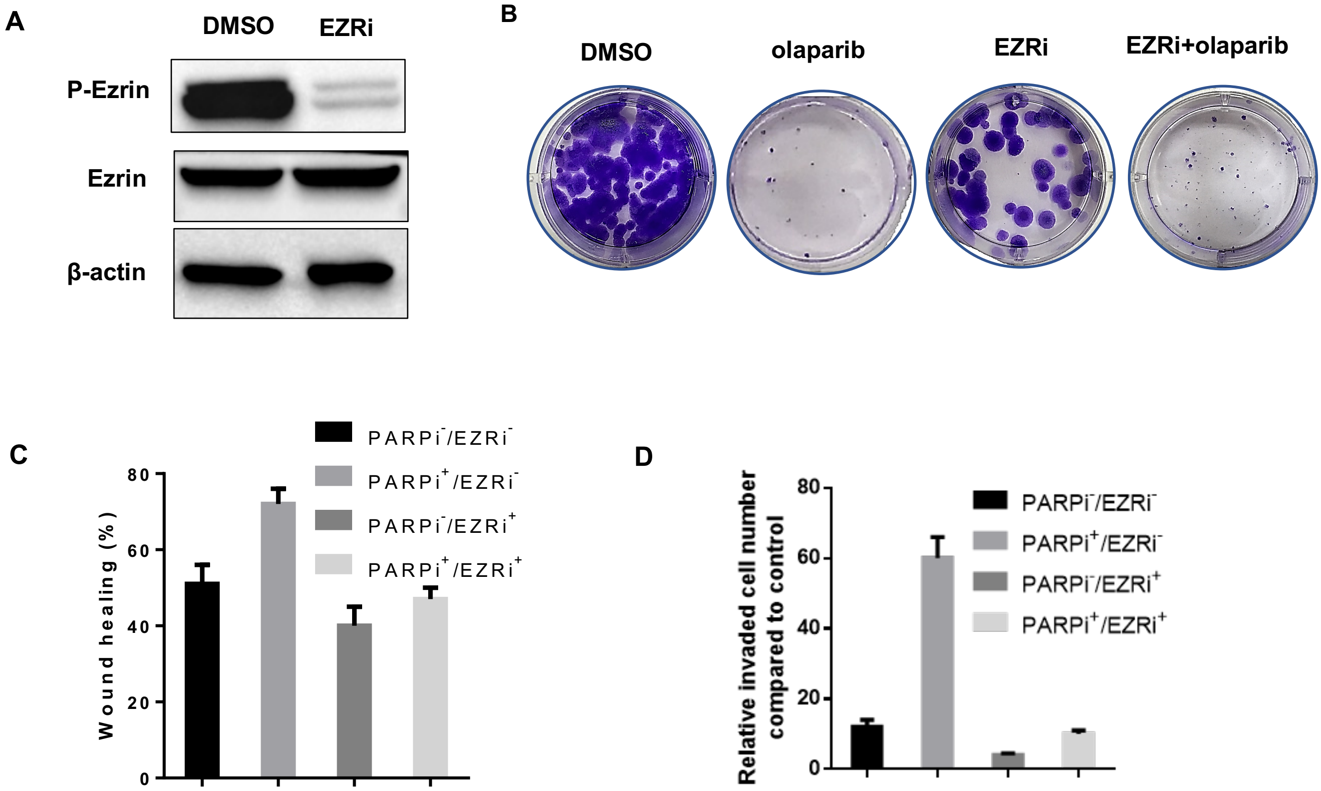

Supplementary Fig 4. Ezrin inhibitor could inhibit proliferation, migration and invasion of OS cells *in vitro*. (A) Western blot analysis of ezrin inhibitor treatment could inhibit the activation of ezrin. **(B)** The proliferation of 143B cells treated with DMSO, ezrin inhibitor, PARP inhibitor, ezrin inhibitor and PARP inhibitor was determined by colony formation assay. **(C)** Wound healing assay (143B cell line) treated DMSO, ezrin inhibitor, PARP inhibitor, or the combination of ezrin inhibitor and PARP inhibitor showed that ezrin inhibition could rescue olaparib induced migration. **(D)** Transwell assay of OS cells treated with or without ezrin inhibitor, PARP inhibitor, or the combination of ezrin inhibitor and PARP inhibitor showed that ezrin inhibition could rescue olaparib induced invasion.
